# From flexibility to flow: psychological selection and the maintenance of values-based therapeutic skills

**DOI:** 10.3389/fpsyg.2026.1967544

**Published:** 2026-10-30

**Authors:** Cedomir Ignjatovic, Antonella Delle Fave, Margaret L. Kern

**Affiliations:** 1 Faculty of Education, The University of Melbourne, Parkville, VIC, Australia; 2 Department of Pathophysiology and Transplantation, University of Milano, Milan, Italy

**Keywords:** contextual behavioural science, flow experiences, lifespan development, psychological flexibility, psychological selection, skill generalisation, wellbeing

## Abstract

Psychological flexibility, the ability to stay present, remain open to difficult thoughts and feelings, and act on personal values, is a well-established transdiagnostic process in contextual behavioural science (CBS) and the principal target of Acceptance and Commitment Therapy (ACT). Yet flexibility skills learned in session often do not last. Homework is frequently left incomplete, skills fail to transfer to the settings where they matter, and a sizeable proportion of clients return to earlier patterns once treatment ends. CBS accounts explain how a flexible repertoire becomes available across settings, but they say less about why a person keeps returning to a specific repertoire rather than another over months and years. To address that gap, this conceptual paper introduces psychological selection, referring to the process by which people tend to preferentially cultivate a subset of the activities available in their daily context, based on the flow experiences that those activities reliably afford. We treat flow as a measurable experiential signal of engagement and absorption elicited by perceived challenge/skill balance and maintained by consequences intrinsic to the activity, and we propose that it can support the sustained practice and refinement of specific flexibility skills after therapy. Whereas psychological flexibility describes the capacity for adaptive responding, psychological selection describes the cultivation of related actions over longer periods of time. We develop an integration model at individual, sociocultural, and therapeutic levels, set out its boundary conditions, including valued action persisting without flow and flow-like absorption that serves no value, and state propositions designed to test whether flow adds predictive value beyond flexibility, values, and reinforcement. Set against habit and rule-governed persistence, our account predicts action retention with growth, repertoires that become more complex as skills rise and individuals search for higher challenge, and it specifies the clinical situations in which the framework does or does not apply. The contribution is a process-level account of how therapeutic gains can be maintained by considering the associated quality of experience.

## Introduction: the problem hiding in plain sight

Contextual behavioural science (CBS), a scientific approach to understanding and influencing human behaviour by focusing on how it is shaped by its context, has identified a robust transdiagnostic mechanism: *psychological flexibility*. It refers to the ability to persist or change behaviour in the service of one’s own values, even among difficult internal experiences. For instance, one might feel intense anxiety before a presentation, but instead of avoiding the presentation, they acknowledge the fear (I am quite anxious about the presentation), connect with their values (I value effective communication), and act (proceeding to give the speech). Psychological flexibility shows consistent associations with mental health across contexts, cultures, and disorders (Hayes et al., 2006; Kashdan and Rottenberg, 2010; Ong et al., 2024). Indeed, meta-analyses indicate that psychological flexibility and inflexibility are associated with wellbeing and psychological distress, respectively, and that psychological flexibility moderates the impact of stressors on mental health outcomes (Gloster et al., 2017).

Acceptance and Commitment Therapy (ACT), the most prominent therapeutic approach developed within CBS, targets this process directly, with meta-analytic evidence showing moderate efficacy across clinically relevant mental and physical health problems (A-Tjak et al., 2015). Recent theoretical advances have further elaborated these foundations through Process-Based Therapy (PBT) and its organising framework, the Extended Evolutionary Meta-Model (EEMM; Hayes et al., 2020; Ong et al., 2024), which applies evolutionary principles of variation, selection, and retention to multiple dimensions of human functioning.

Despite the benefits of psychological flexibility identified by research, relevant skills learned in structured therapeutic contexts frequently fail to generalise to the naturalistic environments where they matter most (Babulal et al., 2016). Homework, the bridge between learning flexibility skills in the therapy room and using those skills in daily life, is often not completed (Kazantzis et al., 2016). Therapists report difficulties with homework in more than half of their cases, and only around two in five clients complete the assigned tasks between therapy sessions (Helbig and Fehm, 2004). Relapse rates provide further evidence for the limitations of symptom-focused interventions that do not explicitly cultivate broad, context-sensitive behavioural repertoires. In substance use disorders, between 40 and 60 percent of people return to active use within a year of treatment (McLellan et al., 2000), with the greatest risk of relapse occurring in the first six months.^1^ Meta-analytic evidence suggests that roughly one-third of clients treated with CBT-based interventions experience recurrence of depressive symptoms (Chen et al., 2022), and 14% experience recurrence of anxiety disorders (Levy et al., 2021). These patterns suggest that the skills learned in structured therapy sessions often fail to generalise to real-world settings, such that individuals revert to well-rehearsed coping strategies when distressed or uncertain, rather than using the skills they are taught during therapy sessions (Babulal et al., 2016).

These statistics highlight a clinical problem, but they do not, on their own, identify a theoretical one. CBS accounts explain much of what happens after a session ends. Relational Frame Theory (RFT) explains how a skill learned in one setting becomes available in another one without direct training, values framed as augmentals explain why an effortful task can take on appetitive functions, and the reinforcement tradition explains how consequences maintain a behaviour once it occurs. What these accounts leave underspecified is the preferential replication of certain learned skills over time. Given the availability of several flexible repertoires consistent with a client’s values, why does the client return to some repertoires and let others lapse across months and years, especially when symptom relief is modest and external validation is scarce? That question is the gap this paper aims to address.

We propose that *psychological selection*, a theoretical construct emerging from the intersection between flow theory and bio-cultural evolution models (Csikszentmihalyi and Massimini, 1985), may represent a complementary mechanism that can contribute to tackling this issue, despite remaining largely ignored by CBS. Psychological selection describes the process whereby individuals throughout their lives tend to preferentially cultivate activities, values, and behavioural patterns based on their association with flow, a gratifying experience of deep absorption, engagement, and autonomous behavioural regulation that arises during tasks subjectively perceived as sufficiently complex to require pervasive skill mobilisation (Csikszentmihalyi and Massimini, 1985; Delle Fave et al., 2011; Massimini and Delle Fave, 2000). Cross-cultural research suggests that at the behavioural timescale, psychological selection operates through the individual’s active and preferential replication of the activities specifically associated with flow experiences. At the developmental timescale, psychological selection contributes to shaping individual development trajectories, creating enduring patterns of preferential resource allocation that transcend immediate contextual contingencies (Delle Fave and Massimini, 2004a, 2005a; Delle Fave et al., 2011, Chapt. 8–9). Where psychological flexibility describes *capacity* for adaptive responding, psychological selection explains *cultivation* of that responding across time and contexts.^2^

We argue that the integration of psychological selection and psychological flexibility as complementary mechanisms may help clarify how therapeutic learning is translated into sustainable behavioural change, by addressing three critical questions that current flexibility models struggle to answer: (1) Why do some clients apply therapy skills consistently while others do not, despite similar gains in flexibility within therapy sessions? (2) How do therapeutic skills become integrated into ongoing behavioural repertoires rather than remaining context-dependent? (3) What processes extend flexibility beyond individuals to social and cultural levels?

## Psychological flexibility: a key mechanism for improved mental health

Psychological flexibility refers to the ability to stay present, open to difficult thoughts and feelings, and act according to one’s personal values despite intervening challenges (Hayes et al., 2006; Kashdan and Rottenberg, 2010). It contrasts with psychological rigidity, which involves an inability to adapt to new information, shift mental gears, or appreciate alternative viewpoints. Psychological flexibility is an important mechanism arising from ACT and involving several key components:

- Present moment awareness: being completely in the here and now, rather than ruminating about the past or worrying about the future.
- Acceptance: remaining open to uncomfortable emotions, thoughts, and physiological sensations, to be present without trying to change or avoid them.
- Values clarity: identifying what matters most across different areas of life, such as family, friends, physical health, work, or education.
- Committed action: acting in accordance with one’s values, despite difficulties that arise in the process.

Through psychological flexibility, individuals acknowledge their thoughts and feelings but, rather than being controlled by them, choose to act in accordance with their values.

Psychological flexibility is related to better physical and mental health, including reduced levels of anxiety and depression (Boykin et al., 2020; Kashdan and Rottenberg, 2010; McCracken and Badinlou, 2021), lower levels of stress (Gloster et al., 2020), less substance abuse (Lee et al., 2015), less chronic pain and improved functioning (McCracken, 2020), higher resilience (Bryan et al., 2015; Leeming and Hayes, 2016; Whiting et al., 2017), and better performance (Bond and Bunce, 2003). In contrast, psychological rigidity relates to higher levels of mental illness (Hernández-López et al., 2021; Ong et al., 2024).

## Psychological selection: the experience-based cultivation of behavioural repertoires

Despite the benefits of psychological flexibility, the translation of skills beyond the therapy room and their generalisation to the range of daily life circumstances and experiences remains a challenge. We suggest that psychological selection offers a complementary mechanism which, when used in conjunction with psychological flexibility-based interventions, might lead to greater real-world impact and reduced relapse rates.

### Theoretical foundations

Csikszentmihalyi and Massimini (1985) introduced psychological selection as an evolutionary concept bridging individual psychology and bio-cultural evolution models. Bio-cultural theories stress the co-existence and interplay of two trans-generational inheritance systems – biology and culture – that shape human behaviours and experiences, fostering the acquisition of new functions, behavioural patterns, and skills to adapt to ever-changing external conditions (Newson et al., 2007; Richerson and Boyd, 1978, 2005). Within this framework, psychological selection emerges from a core proposition: humans rely not only on biological inheritance and passive social learning to acquire adaptive behavioural patterns; they also intentionally select and cultivate a limited subset of the activities, values, and opportunities for skill development available in the environment. They may also actively contribute to their cultural inheritance system through innovation and the creation of new information (Jablonka and Lamb, 2020).

The psychological selection process operates at the intersection of biological predispositions and cultural affordances, with moment-by-moment experiences of perceived optimal challenge, engagement, and autonomous goal pursuit serving as the selection criteria (Massimini and Delle Fave, 2000). This bio-cultural evolutionary perspective anticipates and aligns with the Extended Evolutionary Meta-Model (EEMM; Hayes et al., 2020), which applies variation, selection, and retention across affect, cognition, attention, sense of self, motivation, and overt behaviour, acting at biophysiological, psychological, and sociocultural levels, for both individuals and groups (Hayes et al., 2020; Ong et al., 2024). In the EEMM, selection is defined in relation to a person’s goals and values, rather than clinical procedures. What psychological selection adds, therefore, is not a missing level of the EEMM, but a sharper account of one part of it. It specifies how subjective experiences can act as selection criteria at the motivational level, shaping which repertoires a person preferentially returns to and elaborates over days, months, and years.

More specifically, the experiential signal at the centre of psychological selection is *flow*—a state arising during activities perceived as optimally challenging, and characterised by focused and effortless attention, deep engagement and absorption in the ongoing task, and autonomous regulation (Csikszentmihalyi, 1975, 1990; Nakamura and Csikszentmihalyi, 2002). Extensive phenomenological research involving diverse samples across countries (Delle Fave et al., 2003; Delle Fave and Massimini, 2004a; Delle Fave et al., 2011), as well as quantitative studies conducted through single-administration scales (Jackson et al., 2008) and repeated real-time assessment procedures, such as the Experience Sampling Method (ESM; Hektner et al., 2007) have refined the understanding of flow by identifying its core constituents (for an overview, see Delle Fave and Bassi, 2023). As an experiential state, flow comprises cognitive, affective, and motivational components that together contribute to its optimal features and distinguish it from other positive states. Core characteristics are summarised in Table 1.^3^ Research has highlighted that perceived challenge-skill balance, clear goals, and unambiguous feedback about one’s ongoing performance represent the proximal conditions that promote flow onset and maintenance; the other six components are structural elements of the flow experience (Kawabata and Mallett, 2011; Nakamura and Csikszentmihalyi, 2002).

**Table 1:** Core characteristics of flow experiences.

Flow characteristic	Description
Challenge-Skill Balance	Perceived challenges of the activity and personal skills in balance and higher than average.
Clear Goals and Immediate Feedback	Unambiguous objectives with moment-to-moment information about performance quality enabling continuous behavioural adjustment.
Deep Concentration	Complete absorption in the ongoing activity, with extraneous thoughts and preoccupations fading from awareness.
Merging of Action and Awareness	Activity becomes spontaneous and effortless; reflective self-monitoring dissolves into direct experience of doing.
Sense of Control	Perceived effortless mastery and confident agency in dealing with task demands.
Loss of Self-Consciousness	Temporary transcendence of ego concerns and social evaluation anxieties, fostering a strengthened sense of self after the experience.
Altered Time Perception	Perceiving time as passing either faster or slower than clock time, reflecting deep absorption and focused attention.
Autotelic Experience	Perception of the ongoing task as intrinsically rewarding and worth doing for its own sake, independent of external evaluation or outcomes.

Adapted from Csikszentmihalyi (1990), Delle Fave et al. (2011, Chapt. 3), Massimini et al. (1987), and Nakamura and Csikszentmihalyi (2002). The phenomenological characteristics represent the subjective experiential qualities that distinguish flow from other positive psychological states.

Flow experiences have been described as ‘psychic attractors’ (Massimini and Delle Fave, 2000) that mark the potential of associated activities to be preferentially replicated. Activities associated with flow draw repeated engagement through the intrinsic reward derived from practising them (‘I do it because I want to and it matters to me’), rather than through external reinforcement, such as social evaluation or the therapist’s instruction. Over time, these activities may become preferentially selected for continued investment, skill development, and integration into one’s identity. Phenomenological and quantitative evidence across countries suggests that this selection process operates bidirectionally and across multiple temporal and developmental scales (Delle Fave and Massimini, 2004a, 2004b, 2005a, 2005b). Within the therapeutic process, the context provides clients with opportunities for action (e.g., doing the homework assigned in session); when these activities are associated with flow, clients may perceive the homework as both challenging and manageable, which may sustain preferential practice over time.

Across days and weeks, the practice of values-based therapy skills may develop into the intentional and selective allocation of time and attention towards these activities, producing meaningful engagement. Over months and years, repeatedly using the skills learned in therapy shapes life trajectories through progressive differentiation and integration of skills, values, meanings, and commitments, contributing to the individual’s development (Delle Fave et al., 2011, Chapt. 8, 11). Phenomenological accounts of flow-based developmental trajectories (Csikszentmihalyi, 1996; Delle Fave et al., 2011) as well as longitudinal research on the association of flow with diverse activities (Arai et al., 2025; Bassi et al., 2022; Salanova et al., 2014) and complex developmental tasks such as parenting (Delle Fave and Massimini, 2004b) suggest that optimal experiences sit within self-reinforcing developmental spirals. Initially experiencing flow in a given activity may promote continued practice, practice builds skill, increased skill enables search for greater challenge, and a renewed balance of challenge and skill is the condition under which flow recurs (Massimini and Delle Fave, 2000). Across such a spiral, investment accumulates in what becomes an increasingly differentiated and integrated behavioural repertoire (Rathunde and Csikszentmihalyi, 2006).

Crucially, flow experience does not necessarily involve pleasant emotions. Rather, it signals optimal functioning—the productive intersection of personal capacities and environmental demands. Affective as well as motivational components of flow may substantially vary according to the associated activities. For example, flow experienced by rescuers during a highly complex intervention primarily recruits attention and cognitive processes, rather than eliciting feelings of joy or cheerfulness (Sartori and Delle Fave, 2014). At work or school, flow implies the paradox of relying at least partially on the controlled regulation of behaviour and extrinsic motivators (Bassi and Delle Fave, 2012; Csikszentmihalyi and LeFevre, 1989; Ignjatovic et al., 2026; Peifer and Zipp, 2019). Considering the intersection of multiple subjective and objective components in the onset of flow, related activities are selected for continued investment not merely through volitional deliberation, but through what William James (1890) termed *selective attention*: the preferential allocation of psychological resources towards experiences that enhance psychological and behavioural complexity and integration.

Considering the ceaseless human/environment interplay, through activities associated with flow experiences, individuals may contribute to shaping their bio-cultural milieu by sharing these activities with other people, introducing innovations into existing activities, and creating new activities. When spread by multiple individuals across groups, these activities may become socially retained and transmitted, creating cultural knowledge and traditions that embody both technical skill and valued ways of being (Csikszentmihalyi, 1996; Delle Fave and Bassi, 2009; Delle Fave et al., 2011).

This bidirectional and multilevel temporal perspective distinguishes psychological selection from the search and replication of momentary pleasurable behaviours. As highlighted by cross-cultural studies (Csikszentmihalyi, 1996; Delle Fave and Zager Kocjan, 2017; Zumeta et al., 2016), flow experience may serve as the entry point through which cultural information can be replicated, created, and ultimately selected and transmitted across generations, linking individual phenomenology to collective patterns of development.

### Psychological selection as an integration process within wellbeing and CBS models

Psychological selection aligns with both wellbeing and CBS models. In the wellbeing domain, it complements *Self-Determination Theory* (SDT; Deci and Ryan, 2000), which describes a motivation continuum ranging from the negative pole of amotivation, through progressively less externally controlled types of extrinsic motivation, to intrinsic motivation. The latter is the highest form of self-determination, characterised by engagement in a behaviour for the sake of it, not driven by survival needs, external pressures, or achievement expectations. The drivers of self-determination are the three basic psychological needs of autonomy, competence, and relatedness; their fulfilment promotes wellbeing (Chen et al., 2015; Deci et al., 2017).

The process of psychological selection frames the fulfilment of these basic needs as the motivational component of flow experiences, orienting the differential long-term investment of individual resources in the associated activities (Delle Fave and Bassi, 2016). While SDT focuses on *why* people engage, psychological selection emphasises *which* activities are selected and *how* they become cultivated over time through their repeated association with flow. Specifically, SDT provides a causal account grounded in basic psychological needs, while the functional-contextual perspective of psychological selection focuses on how need-satisfying engagement can be predicted and oriented through contextual opportunities. We treat these as complementary readings of the same phenomenon, and we draw on SDT for its account of intrinsic motivation.

In the CBS context, and specifically in ACT, psychological selection offers a descriptive account at the developmental timescale of how repeated flow experiences in specific value-related activities may support the pursuit of these values, understood as freely chosen life directions that provide meaning and purpose. Values clarification helps identify directions; through the process of psychological selection, and based on their specific biopsychosocial conditions, individuals preferentially pursue some directions, relying on the quality of their experience as an inner “compass” orienting their path (Delle Fave, 2007; Delle Fave and Bassi, 2016). This converges with an account that ACT already offers: valued activity is believed to become rewarding through engaging in the behaviour itself, rather than only through its separate outcomes. Further, values work often aims at lending appetitive, approach qualities to behaviour that would otherwise feel effortful or aversive (Plumb et al., 2009; Wilson and DuFrene, 2009). Psychological selection adds that the quality of experience arising during value-consistent activities is part of what sustains their priority over time.

Clarifications are required concerning three conceptual tenets of the proposed framework. Firstly, it is normatively neutral. Values are chosen, verbally constructed directions, and their authority comes from both the person’s choice and the communities recognising them (Plumb et al., 2009; Wilson and DuFrene, 2009). Flow experience does not define that authority, as some associated activities may serve no value, or serve a harmful one. The proposed framework does not dictate which values are worth holding; it rather describes how chosen directions are sustained, leaving the choice of direction to the person and the clinician’s ethics. The moment-by-moment quality of experience is part of how some chosen directions come to be pursued more than others over time. In therapy, flow is not used to choose or rank a client’s values but rather informs the route to a value. When the quality of experience and a stated value point in different directions, the framework treats this as information about the activities through which the value is being pursued.

Secondly, flow is described as a selection signal, intended neither as an independent causal force nor merely as a metaphor for repeated behaviour. Rather, the term is used descriptively and functionally to identify the experiential pattern associated with activities that individuals preferentially return to, elaborate, and integrate into their behavioural repertoires over time. The conditions facilitating flow onset – perceived challenge/skill balance, clear goals, and immediate feedback – are properties of the relation between person and activity, not of skills alone. For CBS readers, these conditions can also be described as motivating operations, contextual variables that alter the momentary value of engagement (Laraway et al., 2003).^4^ What the quality of experience contributes is therefore selective rather than causal. Moreover, the intentional replication of flow activities over time, with the dynamic interplay between progressive search for higher challenges and related skill refinement (Csikszentmihalyi and Massimini, 1985; Massimini and Delle Fave, 2000), may entail persistent practice of valued activities even during occasions or periods when flow does not occur, a persistence that ACT would describe as values-based and verbally regulated. Causal accounts may instead derive from the behavioural tradition on which CBS builds, using *reinforcement* to explain how consequences can shape behaviour. Positive reinforcement occurs when an individual is rewarded for engaging in a desirable behaviour. Negative reinforcement occurs when behaviour is strengthened by the removal or avoidance of aversive conditions. Flow research describes certain activities as autotelic: their practice is rewarding in itself, independent of external outcomes (Csikszentmihalyi, 1975, 1990). The nearest description in behavioural terms is that these activities are maintained by consequences intrinsic to their practice, rather than delivered by others or by derived material gains (Vaughan and Michael, 1982; Vollmer, 1994).

Thirdly, flow is treated as an empirically measurable feature of person–activity interaction that indicates when an activity is being experienced as intrinsically engaging. These experiential qualities may help orient continued investment in particular activities, but they do not replace contextual, behavioural, motivational, or relational accounts of why these activities are performed. Psychological selection describes how the quality of lived experience is associated with the preferential cultivation and retention of behavioural repertoires across time, while leaving the causal mechanisms of behaviour open to investigation through functional, contextual, and longitudinal methods.

Psychological selection and CBS accounts are therefore better read as complementary levels of analysis of the same phenomenon. Psychological selection is proposed to help explain why clients do or do not maintain skills learned in therapy within their everyday lives over time. Accounts that look only to socially delivered rewards struggle to explain persistent practice of therapy skills despite adverse life circumstances or limited external validation. If therapy skills become vehicles for flow experiences, via mastery of challenging defusion exercises, meaningful engagement in committed action, or values-guided behaviours, they may be selected and cultivated based on their experiential quality, independent of immediate symptom reduction or external validation. This aligns with findings suggesting that optimal experience quality predicts long-term behavioural patterns across diverse contexts, even when those patterns diverge from socially prescribed success pathways (Delle Fave and Massimini, 2004a, 2005b).

## Integrating psychological selection and psychological flexibility: a process-based framework

We suggest that psychological flexibility and psychological selection represent complementary processes that, when integrated, provide a more complete account of how therapeutic skills become sustainably maintained across complex life contexts. We develop this integration across the individual, social/cultural, and therapeutic levels. We particularly focus on the contributions provided by flow research across cultures and developmental stages to enrich the understanding of each level. This multilevel structure aligns with systems-informed positive psychology, which situates wellbeing processes within the broader systems in which individuals are embedded (Kern et al., 2020).

A central role in this integrative view is played by Relational Frame Theory (RFT), which offers the contextual behavioural account of how learning carries from one setting to another. RFT treats human language and cognition as learned relational responding, and it shows how the functions of one event can transform the functions of others related to it in a person’s verbal network (Hayes et al., 2001). A client who learns something in session can have the meaning of an everyday situation shift without direct training, and verbal rules and values come to guide behaviour across contexts. Relational framing also bears on why an activity is worth returning to, through the role of a stated value as an augmental, a verbal cue altering how reinforcing an activity is (Hayes et al., 2001; Plumb et al., 2009), which partly explains why flow tends to arise in valued, engaging, and skill-mobilising tasks rather than at random (Inkinen et al., 2014; Kawabata and Mallett, 2011). Within a single episode, such framing can also shape the appraisal of challenge and skill from which flow arises, since both enter the experience as perceived rather than objective quantities (Csikszentmihalyi, 1990; Delle Fave et al., 2011). We take this episode-level account as a premise here. However, RFT says less about why a person keeps returning to one available repertoire rather than others over months and years. That is the question psychological selection addresses, answering it through the quality of experience that engaging and skill-mobilising activities afford.

Taken together, as illustrated in the *Capacity-and-Cultivation Model* (Figure 1), RFT supplies the reach of a repertoire, while flow experiences account for its preferential cultivation. Psychological flexibility provides the capacity for adaptive responding and thus generates variation in valued, skilful action. Flow during that action works as a selection signal, with proximal conditions acting as motivating operations, and selected repertoires being retained and replicated through consequences intrinsic to the activity itself. Open, flexible engagement supports flow, and flow marks which flexible repertoires are consolidated. Selection marks the preferential replication and meaning development of flow activities, in which flow is maintained over time through progressively increased challenges and skills. The variation, selection, and retention shown in the Figure correspond to the same three principles in the EEMM (Hayes et al., 2020). The upper band shows the sociocultural context acting on the process rather than sitting beneath it, through the availability of activities, their recognition and reward, and their transmission (Kern et al., 2020; Massimini and Delle Fave, 2000; Richerson and Boyd, 2005). At the behavioural timescale, flow arises within a single episode of engagement and marks the activity for replication. At the developmental timescale, repeated replication shapes which repertoires are kept across treatment, follow-up, and the life course, and the therapeutic level is the treatment and maintenance segment of that span. Two cases that the figure does not draw as separate paths belong to the model nonetheless. Flow can mark an activity that serves no value or a harmful one, and the same selection process then works against the person, which is the boundary condition stated below in the fifth proposition. Further, valued action can be sustained without flow, through values framed as augmentals, committed action, and social contingencies, as described below in the sixth proposition. The Boundary Conditions subsection sets out where each case applies.

**Figure 1:**
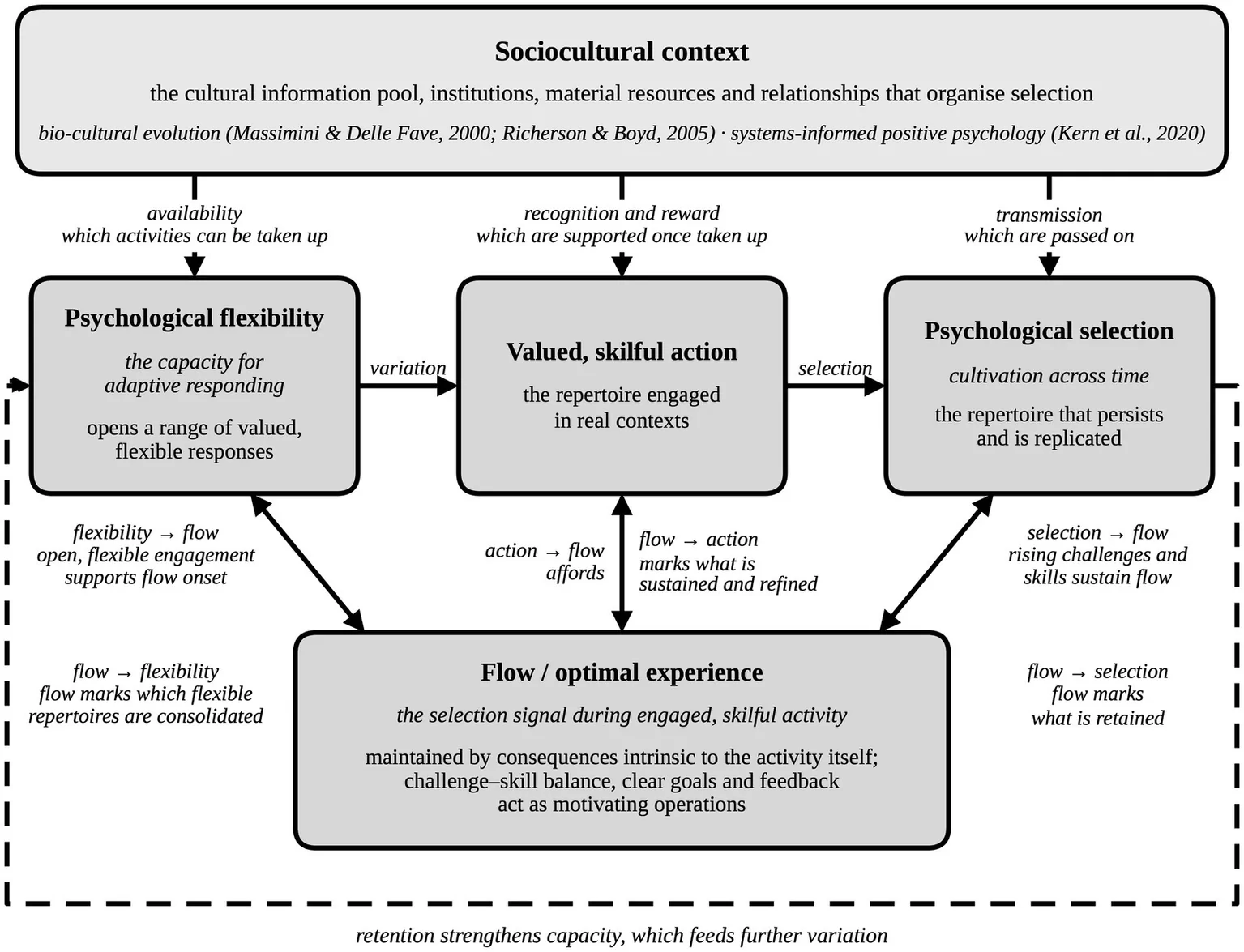
The Capacity-and-Cultivation Model. The model does not imply that flow is necessarily values-consistent or necessary for valued action; these boundary conditions are specified in Propositions 5 and 6.

The bidirectional arrows in Figure 1 reflect the proposal, consistent with evidence on creativity and flow, that these processes feed into one another rather than act as a one-way pipeline. Flexible engagement with a task supports problem finding and the detection of engaging opportunities for action, while flow in turn marks the flexible repertoires that are consolidated (Csikszentmihalyi, 1996; Delle Fave and Kawabata, 2026). Valued action and flow likewise sustain each other: engaging and skilful activity facilitates flow, and recurring flow experiences contribute to refining and maintaining action. Notably, many clients may have experienced flow during daily life activities before entering therapy, so the therapist can build on an existing experiential repertoire rather than introducing an unfamiliar state. Moreover, as psychological selection and flow are mutually sustaining through the gradual increase of related challenges and skills, identifying life activities or domains to which clients repeatedly return could help support their behavioural complexity, by embedding valued action within their flow-based developmental trajectory.

### The specific contribution of psychological selection

At this point, a reader working within CBS might still ask what psychological selection adds to psychological flexibility, relational framing, and process-based models. We suggest three additions, which the propositions described below depend upon.

First, the psychological selection framework allows for observing flow-based activity practice and replication as it unfolds, rather than inferring it afterwards. In the behavioural tradition, reinforcement is identified by its effect on future behaviour frequency, such that an activity is known to have been selected only once it has been repeated. Flow experience can be identified during activity practice using ESM. Our framework suggests that it predicts which repertoires will be retained. If our prediction holds, clinicians gain a real-time indicator of maintenance during treatment rather than a *post hoc* indicator at follow-up.

Second, the flow literature describes a trajectory that a reinforcement account does not provide on its own. Continued engagement in an activity depends on perceived challenges increasing as skills are progressively refined; when this dynamic rebalancing does not occur, engagement tends to lapse into boredom (Csikszentmihalyi and LeFevre, 1989; Delle Fave et al., 2011). This describes why repertoires grow more complex over time and gives the framework a prediction about how values-based behavioural repertoires are cultivated, not merely whether they occur.

Third, the framework separates repertoires maintained by consequences intrinsic to the activity from repertoires maintained by external consequences. This classical distinction in behaviour analysis (Vaughan and Michael, 1982; Vollmer, 1994) is rarely measured in clinical work, despite its key role in explaining the homework problem. A skill practised for the sake of the therapist’s approval versus a skill practised by virtue of its engaging structure may look identical within the session but manifest differently once the session ends. Taking flow into account creates a sense of co-creation between the client and the therapist in maintaining values-based behaviours over time.

These additions lead to predictions about maintenance that reinforcement, motivation, and values do not make on their own. A reinforcement account identifies a consequence as reinforcing by its effect on the future rate of behaviour, so whether—and through which consequences—a skill has been maintained can be established only after repetition or lapse has occurred. During treatment, several consequences operate at once, including the therapist’s approval, the homework review, symptom relief, and the structure of the activity itself, and the account does not by itself say in advance which of them will still be operating when the sessions end. Flow is proposed as a marker of the last of these—the consequences intrinsic to the activity—which may continue to operate beyond therapy. We predict that, among skills receiving comparable therapist-mediated and other external reinforcement during treatment, those practised while experiencing flow will be more likely to be retained after discharge than those practised without flow (Propositions 2 and 3).

Accounts of intrinsic motivation explain persistence in terms of a person’s reasons for engaging in an activity, often assessed at the person or activity level, whereas flow is a momentary state that can fluctuate across episodes of the same activity as perceived challenge and skill change. Our within-person prediction, specified in Proposition 3, is that flow during one episode will predict the likelihood of subsequent practice beyond the person’s more general motivation for the activity.

Values predict the direction of behaviour and its persistence under aversive conditions, as reflected in Proposition 6. However, many different activities can be consistent with the same value, and values alone do not say which of them a person will keep. We predict that, among activities equally consistent with a stated value, those that reliably afford flow experiences will be more likely to be retained and elaborated, whereas otherwise comparable activities may be less likely to persist once explicit therapeutic prompts or commitments are withdrawn (Propositions 3 and 6). In each case, the claim is incremental rather than competing. Flow discriminates within the set of behaviours that reinforcement, motivation, and values already predict will be maintained, and each of the three predictions can fail.

These additions may be particularly relevant to the maintenance issue, which is especially important to clinicians. Within CBS, habit can explain behavioural persistence by context-cued repetition, and it grows more automatic with each repetition (Lally et al., 2010; Wood and Rünger, 2016). Rule-governed persistence can explain it through verbal control, and behaviour under rule control tends to stay insensitive to changing consequences (Hayes et al., 1986). A repertoire retained through psychological selection follows a third pathway. Because continued engagement depends on seeking progressively higher perceived challenges as skills are refined, the repertoire should become more differentiated and integrated over time, signalling the growth in complexity described by flow research since its foundational works (Csikszentmihalyi, 1975, 1990). On this account, retention with growth is the signature of psychological selection, and it is the prediction that distinguishes it from habit and rule-following. Indirect evidence consistent with components of this prediction comes from Csikszentmihalyi’s biographical interview work with artists and scientists (Csikszentmihalyi, 1996), ESM findings showing positive associations between flow-related activities and perceived long-term goals (Delle Fave and Massimini, 2004b, 2005a) and mixed-method studies investigating flow together with perceived present challenges and future goals across cultures (Delle Fave et al., 2011, Chapt. 7), among persons with disabilities (Delle Fave and Massimini, 2004a, 2005b) and among migrants (Delle Fave and Bassi, 2009).

### Individual level: flow as a selection signal for flexible responding

At the individual level, psychological selection provides a pathway through which flexible responding becomes preferentially strengthened and maintained. Consider a client learning defusion in ACT-based therapy. Traditional accounts emphasise how defusion reduces the behaviour-regulatory impact of difficult thoughts (Hayes et al., 2006).^5^ This explains *capacity*—why the client *can* respond more flexibly when previously fused with self-critical thoughts. But it does not fully explain flexible response *cultivation*—why the client continues using defusion weeks and months after therapy ends, particularly when immediate symptom relief is modest or when life circumstances are demanding.

Psychological selection offers a complementary explanation grounded in the quality of experience during the use of therapy skills. For instance, when defusion is practised with a level of challenge the client subjectively perceives as appropriate—neither rote mechanical application nor overwhelming burden—we propose that it can afford flow experiences (P1, P2). The experiential profile of flow—heightened focus on the task and fading of rumination, self-criticism, and ego-involvement (Ulrich et al., 2016)—overlaps with what defusion is meant to produce. The clear and immediate feedback regarding the effects of the therapeutic technique on thought-behaviour relations would support the merging of action and awareness. As the skill becomes more spontaneous, the person gains a sense of control over their psychological processes and experiences intrinsic motivation in mastering them.

### Social and cultural level: collective flow experience as flexibility transmission

Psychological selection extends beyond individuals to social and cultural levels, which sit at the centre of bio-cultural frameworks (Delle Fave and Bassi, 2016; Massimini and Delle Fave, 2000). The EEMM already includes a sociocultural level. Group-based applications such as Prosocial work directly with cooperation and the cultural practices that shape behaviour, and DNA-V extends the same model to development and social connection in young people (Atkins et al., 2019; Hayes and Ciarrochi, 2025; Hayes et al., 2020). What flow research adds is the dynamics supporting the replication and dissemination of some shared activities and not others, centred in the quality of experience they reliably afford. Cross-cultural evidence suggests that optimal experiences are not randomly distributed, but cluster around culturally elaborated activities that communities have refined over generations. These activities—including traditional crafts, cooperative work practices, artistic performances, and community rituals—embody collective wisdom about how to structure challenge, provide feedback, and support skill development in ways that are reliably associated with the phenomenological characteristics of flow (Delle Fave et al., 2003; Delle Fave and Massimini, 2004a, 2005b; Delle Fave et al., 2011, Chapt. 12; Delle Fave and Zager Kocjan, 2017).

Two features need to be acknowledged at the sociocultural level. First, psychological selection at the individual level operates within a context already structured by culture. The local cultural pool and the institutions that hold it determine which activities are available to be selected, which are recognised and rewarded, which are sanctioned, and which are transmitted to the next generation (Massimini and Delle Fave, 2000; Richerson and Boyd, 2005). Second, access to these activities is unequal. Studies of optimal experience across cultures and among migrants, disadvantaged minorities, and people living with disability or suboptimal health conditions show that flow can be achieved under severe constraint, but also that material resources, social recognition, and institutional arrangements shape which activities a person can cultivate and for how long (Delle Fave et al., 2003; Delle Fave and Bassi, 2009; Delle Fave and Massimini, 2004a, 2005b). The sociocultural context therefore shapes selection rather than merely hosting it, by setting the supply of activities, the contingencies attached to them, and the pathways by which they are passed on. This makes the proposed framework necessarily multilevel and converging with systems-informed positive psychology, which places wellbeing processes within nested social systems (Kern et al., 2020).

This cultural dimension has direct implications for psychological flexibility. When therapy skills become embedded in activities that the client’s community already recognises as flow-producing, we propose that those skills gain additional selection advantages (P4). Group ACT interventions that incorporate shared challenging activities—such as experiential exercises, values-based community projects, or collective meaning-making practices—may leverage this collective selection process. This would occur through both the promotion of flow experiences and the strengthening of individual skill development and social bonds, thus creating social empowerment through personal growth (Delle Fave and Massimini, 2005b; Delle Fave et al., 2011, Chapt. 7, 14).

Moreover, ACT interventions designed to connect flexibility skills to culturally valued activities that naturally incorporate the structural conditions of flow – challenge/skill balance, clear goals, and immediate feedback – may enhance both initial engagement and long-term maintenance. This represents a shift from viewing therapy as individual skill training to a process facilitating individuals’ connection to culturally elaborated practices that sustainably support flexible responding through their intrinsic experiential qualities.

### Therapeutic level: designing for experiential selection

At the therapeutic level, integrating psychological selection with flexibility suggests concrete modifications in conceptualising and implementing interventions. Besides building capacity for flexible responding, during therapy skill acquisition and values-based action therapists might encourage the identification and cultivation of flow-related activities. We suggest that the dual focus on psychological selection and flexibility may address the previously identified homework compliance and generalisation issues. On this account, we propose that skills whose practice is reliably associated with flow will be more likely to be preferentially enacted, refined, and generalised (P3); the quality of experience represents the marker by which they can be identified during treatment.

A comprehensive review of flow assessment tools (Delle Fave and Bassi, 2023) provides methodological guidance for therapeutic application. Repeated real-time assessment procedures, such as ESM (Hektner et al., 2007), could help identify which homework activities are prominently associated with flow and which ones are primarily connected with other experiences, such as boredom, anxiety, or apathy. The same procedure can map the daily life activities in which a client already experiences flow before entering therapy, such that new therapy skills can be anchored to existing sources of flow experience. As an illustration rather than evidence, a client who surfs most mornings and is learning to defuse from catastrophic thoughts has, in surfing, an activity with clear goals, immediate feedback, and a challenge that can be increased based on skill refinement, and that requires deep concentration, absorption, and lower self-observation, especially in challenging sequences and conditions. Defusion practised through surfing-related flow occurs without a homework sheet. The value that makes surfing worth doing comes from the client; the experiential quality of surfing may help sustain the activity through which that value is enacted. This information may orient the design of individualised homework assignments that optimise challenge-skill balance, providing sufficient difficulty to maintain engagement without overwhelming the client’s capabilities. Importantly, this balance is not static and must be continuously adjusted as skills develop, suggesting the need for ongoing assessment and homework modification throughout treatment (Delle Fave and Bassi, 2016).

Attending to how a therapy skill is experienced is already part of good ACT practice, when clinicians ask clients to notice the texture of a present-moment exercise or the felt quality of values-based action. What flow research adds is a more precise vocabulary for that noticing, a range of experiences (e.g., flow, anxiety, boredom, apathy) to be considered, and a way to assess them. Therapists might also explicitly discuss with clients the experiential quality of different flexibility skills, using phenomenological language drawn from flow research. For instance, during defusion practice, a therapist might ask: ‘To what extent have you experienced a sense of effortless concentration, as if your attention were naturally flowing with the practice?’ Or ‘During committed action, to what extent do you feel fully absorbed in what you are doing, forgetting all concerns? On these occasions, do you notice time passing differently?’ Such questions may make the quality of experience a legitimate therapeutic focus, potentially enhancing clients’ awareness of which therapy skills reliably produce flow experiences in activities worth cultivating. They align with the phenomenological approach often adopted in flow-related studies, as well as the findings obtained from research conducted in clinical contexts (Bassi et al., 2012; Delle Fave et al., 2011; Sartori et al., 2014).

Furthermore, based on evidence showing that values alignment with flow-producing activities is associated with sustainable behavioural patterns (Delle Fave and Massimini, 2005b; Delle Fave et al., 2011), a client’s values clarification might be enriched by explicitly exploring which life domains and activities have previously been, or are currently, associated with flow experiences. A client who consistently experiences flow in creative activities but identifies career advancement as a primary value might benefit from exploring whether their career path incorporates creative challenge and, if it does not, where within that path creative challenge could be built in (Yan and Donaldson, 2022). The value stays where the client has put it. Flow informs the route, such that the path towards the value incorporates activities with structural features that support flow and thereby psychological selection.

### Boundary conditions

There are several boundaries to our framework. First, it is specifically focused on the maintenance and relapse-prevention phase of treatment, rehabilitation, and return-to-work planning, when the client is or can be engaged in a structured activity potentially associated with flow, and when the clinical task has shifted from acquiring flexibility to maintaining flexible responding. Homework design becomes relevant once safety and stabilisation are in place. Everyday coping associated with suboptimal experiences lies outside the claim. The research evidence of flow occurrence in psychiatric and neuromotor rehabilitation (Bassi et al., 2012; Sartori et al., 2014) and in unfavourable contexts (Delle Fave et al., 2003; Delle Fave and Massimini, 2005b) suggests that the approach is not restricted to affluent societies and healthy individuals. What changes across settings is the place of flow in the sequence of care rather than the framework’s general applicability.

Second, our framework does not speak to circumstances where the client’s conditions are unsafe or unstable, basic needs are unmet, or the presenting problem is acute trauma, displacement, or ongoing existential insecurity. Under these circumstances, the primary goal is safety, stabilisation, and trauma treatment. Nevertheless, the availability and practice of flow-producing activities may offer a temporary relief, secondary to the main clinical intervention (Burt and Tosado, 2025). Intentional flow-based selection may become relevant later, as a way of deciding which of the available activities are worth cultivating.

Third, the framework does not apply to conditions where the client has no access to structured activity, or where severe anhedonia removes the experiential signal that the framework depends on. In those cases, values-based persistence is the relevant route.

## Core propositions and hypotheses

We advance six process-level propositions (P1 – P6) that make the integration of psychological flexibility and psychological selection generative and testable. Table 2 summarises each proposition with its rationale, the pattern it predicts, how it could be tested, the constructs that would need to be controlled, and the evidence that would count against it.

**Table 2:** Propositions and testable implications.

Proposition	Theoretical rationale	Predicted pattern	Design and measures	Constructs controlled	Evidence that would count against it
P1. Psychological flexibility increases behavioural variation and so exposure to the conditions that support flow.	Present-moment and acceptance processes free attention for the task; flexible engagement supports problem finding and the pursuit of valued challenge. Mindfulness is moderately associated with flow (Schutte and Malouff, 2023).	Momentary flexibility, particularly present-moment and acceptance processes, relates to a higher probability of flow during values-based action.	Experience sampling across episodes of values-based action; momentary flexibility items alongside a state flow measure (Delle Fave and Bassi, 2023; Jackson et al., 2008); within-person analysis.	Perceived task challenge and skill, existing skill level, positive affect at the start of the episode.	No within-person association between momentary flexibility and flow once affect and skill are controlled.
P2. Flow during skill practice marks skills maintained by consequences intrinsic to their use and carries part of the effect of activity structure on completion.	Perceived clear goals, feedback, and calibrated challenge act as motivating operations for engagement. The experiential quality of practice is proposed to carry part of the effect of structure on completion rather than structure acting directly alone.	Momentary flow mediates the effect of flow-supportive activity structure (challenge–skill calibration, clear goals, immediate feedback) on homework completion and real-world skill use.	Prospective design across homework occasions; structure and expectations rated at assignment, flow at practice, completion and use at the next session; mediation tested with the temporal order preserved.	Perceived usefulness of the exercise; therapist-delivered reinforcement.	The structure effect on completion is unchanged when momentary flow is removed from the model, or flow is unrelated to completion once usefulness is controlled.
P3. Repeated flow during values-based action is associated with retention through progressive skill refinement and identity integration.	Activities that reliably afford flow are preferentially replicated; replication builds skill and fosters search for higher challenge; the activity becomes part of the person’s repertoire and self-description.	Flow during homework prospectively predicts next-day repetition and follow-up maintenance.	Experience sampling during treatment with daily repetition records; follow-up at six and twelve months with behavioural indicators of skill use.	Baseline psychological flexibility; values-consistency of the activity; reinforcement-based predictors; symptom change.	Flow predicts repetition only through its overlap with values-consistency or reinforcement, or does not predict follow-up maintenance once these are controlled.
P4. At the sociocultural level, shared flow in culturally acknowledged activities relates to the dissemination and maintenance of flexible repertoires over and above individual experience.	The cultural pool and its institutions set which activities are available, recognised, rewarded and transmitted; access is unequal; settings differ in the challenge, feedback and social contingencies they arrange.	Retention of a flexibility skill varies with the structural properties of the activity and setting and with social contingencies for continued participation. Embedding an intervention in a community-recognised activity yields higher engagement and maintenance than individual skill training.	Multi-site or multi-setting comparison with setting characteristics coded; cluster-level comparison of embedded versus individual delivery.	The person’s own flexibility, values-consistency and momentary flow.	Setting effects disappear once individual flow is controlled, or embedded and individual delivery do not differ in maintenance.
P5. Flow is not inherently values-consistent; values clarity and flexibility moderate whether flow-based selection supports wellbeing or entrenches maladaptive persistence.	Absorption and time distortion also arise in rigid or compulsive patterns; old repertoires selected on experiential grounds compete with newly learned skills at the developmental timescale.	The flow–outcome association is moderated by values-consistency and flexibility; where the old repertoire carries its own experiential pull, relapse risk is higher when the new repertoire has not become a source of flow.	Moderation tested in treatment samples with flow, values clarity and flexibility assessed; relapse followed after discharge in populations with gambling, gaming, substance use or other addictive behaviours.	Symptom severity at discharge; baseline flexibility; values-consistency of the new repertoire.	High absorption predicts positive outcomes regardless of values clarity, or relapse risk is unrelated to whether the new repertoire affords flow once severity and flexibility are controlled.
P6. Valued action can be practised in the absence of flow; flow adds predictive value when perceived challenge/skill calibration and clear goals are available within the activity.	Early skill acquisition and exposure-based work are carried by values framed as augmentals, committed action, and social contingencies; flow conditions are rarely present until skill has developed.	The association between momentary flow and subsequent repetition is weak or absent early in treatment and strengthens as skill develops, whereas values-consistency predicts persistence throughout.	Experience sampling across the course of treatment with perceived challenge and skill levels rated; time-varying association between flow and repetition modelled against values-consistency.	Perceived challenge and skill levels; stage of treatment; social contingencies for practice.	Flow predicts repetition as strongly early as well as late, or values-consistency stops predicting persistence once flow is available.

All propositions are hypotheses for empirical test rather than established findings. The cited evidence supports the component claims drawn from flow research and contextual behavioural science separately; no study has yet tested the propositions as stated.

*P1*: First, we propose that psychological flexibility increases behavioural variation by enabling the pursuit of valued challenges, thereby increasing exposure to conditions that support flow experiences. Meta-analytic evidence supports this view, suggesting that mindfulness, understood in ACT as encompassing a broader set of processes, including present-moment awareness, acceptance, and defusion, is moderately associated with flow (Schutte and Malouff, 2023). Consistent with this mindfulness-flow association, we hypothesise that momentary psychological flexibility, particularly present-moment and acceptance processes, will relate to a higher probability of flow experiences during values-based action.

*P2*: Second, we propose that when flexibility skills are practised under flow conditions (Table 1), the associated experience acquires flow characteristics; this experiential quality may mark skills that are being maintained by consequences intrinsic to their use. We hypothesise that momentary flow during skill practice will mediate, in part, the relationship between flow-supportive activity structure—particularly challenge–skill calibration, clear goals, and immediate feedback—and subsequent homework completion and real-world skill use. Although activity structure alone can improve completion (Kazantzis et al., 2016), the specific prediction is that the experiential quality of practice carries part of that effect, and that it continues to do so once the perceived usefulness of the exercise and therapist-delivered reinforcement are controlled.

*P3*: Third, we propose that repeated flow experiences during values-based action are associated with activity retention through progressive skill refinement and identity integration, increasing the probability that flexibility skills will generalise across contexts and persist after treatment. We hypothesise that flow experiences during homework exercises will prospectively predict next-day repetition and follow-up exercise maintenance above and beyond baseline psychological flexibility, values-consistency of the activity, and reinforcement-based predictors, and not only above and beyond symptom change.

*P4*: Fourth, at the sociocultural level, we propose that shared flow in culturally acknowledged activities may relate to the dissemination and maintenance of flexible repertoires through these routes, over and above individual experience alone. Specifically, we hypothesise that the probability that a flexibility skill is retained will vary with the structural properties of both available activities and the practice setting, as well as with the presence of social contingencies for continued participation, above and beyond the person’s own flexibility, values-consistency, and momentary flow. Further, we hypothesise that embedding an ACT intervention in an activity already recognised by the local community may promote higher initial engagement and longer-term maintenance compared to an equivalent intervention delivered as individual skill training, with the difference carried by access, recognition, and transmission, over and above flow features.

*P5*: Fifth, flow is not inherently values-consistent. High absorption and flow-like states can also arise during the initial practice of rigid or harmful patterns (e.g., gambling or disordered gaming), implying that values clarity and flexibility should moderate whether flow-based selection supports wellbeing or entrenches maladaptive persistence and experiential drift towards obsessive or dependent patterns (Delle Fave et al., 2011, Chapt. 15; Dixon et al., 2019; Footitt et al., 2024; Kruger et al., 2022; Vallerand, 2008). We hypothesise that the association between flow and positive outcomes is moderated by values-consistency and flexibility, such that when values clarity or flexibility are low, high absorption may predict maladaptive persistence. At the developmental timescale, this proposition informs relapse. The well-rehearsed strategies to which clients return often include repertoires that were selected on experiential grounds long before treatment began. A newly learned flexibility skill may compete with them. Where the old repertoire carries its own experiential pull through maladaptive activities, such as gambling, excessive gaming, and substance abuse, we predict that relapse risk after treatment will be higher when the new repertoire has not itself become a source of flow, over and above symptom severity at discharge, baseline psychological flexibility, and the values-consistency of the new repertoire.

*P6*: Sixth, valued action may be practised in the absence of flow. Clinically meaningful behaviour is often effortful, repetitive, and experientially difficult, and in the early stages of change a person may practise it only because it serves a value. The Capacity-and-Cultivation Model presented here does not require that a behaviour become intrinsically engaging in order to persist; rather, early skill acquisition and persistence in exposure-based work can be carried by values framed as augmentals, committed action, and social contingencies, with little contribution from flow. Flow begins to add predictive value once the flow conditions – in particular, perceived challenge-skill calibration and clear goals – are satisfied within the activity. We therefore hypothesise that the association between momentary flow and subsequent repetition will be weak or absent early in treatment, and strengthen as skill develops, whereas values-consistency will predict persistence throughout.

## Empirical implications and future directions

The integration of psychological selection and psychological flexibility offers several research directions, with predictions and expected patterns summarised in Table 2.

### Assessment and measurement innovations

A starting point could be the joint evaluation of the client’s flexibility and flow experiences. As highlighted by Howell and Demuynck (2021), flexibility/inflexibility levels (measured through the Multidimensional Psychological Flexibility Inventory, MPFI; Rolffs et al., 2018) show strong correlations with scores on the Questionnaire for Eudaimonic Well-Being (QEWB; Waterman et al., 2010). Notably, several QEWB items refer to key dimensions of flow, such as present moment awareness, perceived self-expression, and committed action.

Several instruments used to assess flow (for a review, see Delle Fave and Bassi, 2023) could be adapted in psychological flexibility research. Momentary assessment procedures such as ESM offer promise for capturing the experiential quality of flexibility skill use in naturalistic settings and in real-time, revealing the extent to which therapy skills are associated with the experiential flow characteristics. Studies conducted in rehabilitation settings (Bassi et al., 2012; Sartori et al., 2014) and during life transitions (Delle Fave and Massimini, 2004b) show the potential clinical relevance of ESM findings for designing long-term treatments. In the clinical context, the feasibility of this approach is supported by a recent ecological momentary intervention prompting clients to practise therapy skills in daily life and record their immediate emotional response and its relation to outcomes (Bernstein et al., 2022). Mixed-method tools, such as the Flow Questionnaire (Delle Fave et al., 2011), as well as scales investigating state and dispositional flow (Jackson et al., 2008), represent other useful sources of information. However, adaptation for therapy contexts requires careful conceptual work to preserve their phenomenological validity while achieving clinical utility.

Specific research questions arising from this approach include the following. Do therapy homework assignments that are more frequently associated with flow experiences show better adherence rates? Does the experiential quality characterising initial skill practice predict long-term maintenance after treatment termination? Are there individual differences in the implementation of flexibility skills through flow, allowing personalised intervention matching? Beyond symptom-focused endpoints, do flow-supported flexibility skills also predict gains in eudaimonic wellbeing over time (Huta and Waterman, 2014)?

Additionally, the investigation of the temporal dynamics that allow for the preferential replication of flow-related activities in the long run requires prospective studies with longitudinal designs incorporating the whole treatment and its follow-ups, to capture both experience fluctuations and behavioural changes over time. For example, it would be important to ascertain whether clients who report flow during values-based action show steeper gains in values-consistent behaviour over follow-ups, or whether experiencing flow during therapy predicts resilience to relapse-inducing stressors. These studies might incorporate both ESM and psychometrically valid questionnaires for immediate effects, as well as mixed-method approaches using questionnaires and semi-structured interviews for longer timescales.

### Intervention adaptations targeting experiential selection

The proposed integration suggests several intervention modifications grounded in flow research. First, homework might systematically incorporate the conditions identified as flow antecedents. Rather than a generic prompt to ‘practise defusion daily,’ assignments might specify concrete and subjectively appropriate challenges that provide unambiguous success criteria (e.g., ‘use leaves-on-a-stream with your three most distressing thoughts about the upcoming presentation’). The same design logic extends to the investigation of daily activities, which can be integrated into the exploration of clients’ psychosocial resources, such as personal strengths use, which has been linked to flow at work and schools (Ignjatovic et al., 2026; Wagner et al., 2020).

Second, we infer from cross-cultural research that culturally adapted interventions aligning flexibility skills with locally valued activities may be more likely to afford flow experiences, although no intervention of this kind has yet been tested. This moves beyond superficial cultural translation towards substantive integration with engaging and meaningful individual and social activities that communities have refined over generations, thereby leveraging collective psychological selection processes.

Third, group formats might be designed to explicitly cultivate shared flow experiences. Research on collective flow (Delle Fave et al., 2003; Pels et al., 2018; van den Hout et al., 2018; Zumeta et al., 2016) shows that coordinated challenging activities (e.g., musical performances, athletic cooperation, collaborative problem-solving) are associated with distinctive experiential qualities and with reports of skill development and social cohesion. Group ACT exercises structured as collective challenges with clear shared goals and moment-to-moment feedback may enhance not only skill acquisition but also the experience-based selection that sustains longer-term practice.

## Discussion

### Conceptual challenges and limitations

The proposed integration has several conceptual and practical challenges and limitations. First and most importantly, the integration itself has not been empirically tested. The flow literature and the CBS literature support the component claims separately, and the six propositions and Table 2 are hypotheses rather than findings. The clinical implications are translational hypotheses that hold only if the second, third and sixth propositions survive the tests set out in Table 2.

Second, the relationship between flow’s phenomenology and functional analysis requires careful specification. Flow theory, rooted in humanistic-phenomenological traditions, emphasises subjective experiences as primary data. CBS, emerging from functional contextualism, prioritises behavioural prediction and influence over phenomenological description. While these perspectives complement rather than contradict each other, their genuine integration requires showing how experiential quality orients and shapes behavioural repertoires through selection processes, rather than correlating with them.

Third, the bio-cultural framework, which positions subjective experience as an evolved mechanism for selecting adaptive behavioural patterns, may support this integration effort. Findings from flow research support this view. This empirical regularity in how people engage in daily activities, momentarily and in the long run, does not overlook or exclude the role of reinforcement.

Fourth, the causal direction between psychological flexibility and flow remains unclear and may even be non-existent. From the systemic perspective we rely upon (Kern et al., 2020), causal claims are superficial and do not capture the complexity of ongoing optimal experiences. Flexibility training could enable flow by removing psychological barriers to optimal challenge-skill engagement; the cultivation of flow in therapeutic activities could build flexibility through repeated practice in optimal experiential contexts; both processes could run through mutually supportive feedback cycles. Reciprocal relationships across development have been suggested in the flow literature exploring creativity and developmental processes (Delle Fave et al., 2011; Delle Fave and Kawabata, 2026; Freire et al., 2021), yet the flexibility and flow relationship has rarely been directly examined. Dismantling designs that compare flexibility training with and without the explicit cultivation of flow, along with mediation analyses examining temporal sequences, would clarify whether and which causal pathways exist.

Fifth, the possibility of long-term selection of maladaptive activities that provide initial flow-like experiences needs to be considered. High absorption and time distortion can occur in rigid or compulsive patterns, where experiential reward may maintain behaviour despite harmful consequences (Dixon et al., 2019; Kruger et al., 2022). Similarly, pronounced loss of time awareness has been linked to elevated risk for disordered gaming (Footitt et al., 2024). We predict that psychological flexibility and values clarity should moderate whether flow-based selection supports valued living or entrenches maladaptive persistence (Delle Fave, 2013; Delle Fave and Massimini, 2005b).

## Conclusion

We have argued that psychological selection provides a complementary mechanism to psychological flexibility that may address persistent challenges in therapy homework compliance and skill generalisation, sustaining the integration of behaviours learned during sessions into ongoing repertoires over months and years.

The integration operates across multiple levels. Individually, we propose that therapy skills that afford flow may be maintained by consequences intrinsic to their use, with experiential quality serving as a marker of their likelihood of retention. Socio-culturally, connecting flexibility training to culturally acknowledged flow-producing activities may leverage collective selection processes that transcend individual therapy. Clinically, designing interventions to optimise experiential quality may enhance both acquisition and maintenance of flexible responding. The process of psychological selection, integrated with the bio-cultural framework, provides theoretical grounding for these claims, while the component claims rest on the flow and CBS literatures separately and the integration itself awaits direct test, linking phenomenological experience to adaptive development across biological and cultural timescales.

Flow is proposed here as a signal of which valued activities are likely to be sustained, in the service of directions the client has already chosen, rather than as an ideal around which a life should be organised. Our framework suggests that interventions can do more than build skills or restructure cognitions. They can also connect clients to activities, practices, and community rituals that reliably afford flow, through their structural features.

While psychological flexibility describes our capacity to orient behaviour towards values despite difficult internal experiences, psychological selection describes the process through which that capacity is expressed, contributing to the course of individual development. This integration thus draws out an aim that CBS already holds – attending to how optimal experiences and the activities that support them are attained and replicated across the lifespan. If CBS aims to create a behavioural science adequate to capture the complexity of human existence, one that includes thriving as well as disorder, then the experiential processes through which behavioural repertoires become cultivated and selected fall within its remit. Psychological selection integrated with psychological flexibility offers one way to bring them together. Psychological flexibility explains the availability of adaptive, values-consistent behavioural variation; psychological selection helps explain why some of those behavioural possibilities are preferentially cultivated and retained over time through their association with flow experiences.

## Data Availability

The original contributions presented in the study are included in the article/supplementary material, further inquiries can be directed to the corresponding author.

## Footnotes

1 How relapse is defined varies across this literature (Maisto et al., 2016; Sinha, 2011) and by clients themselves, so these figures are best read as a return to earlier use rather than as a single endpoint of relapse.

2 A terminological note is needed here, because the two traditions use the word “skill” differently. In ACT and other therapies grounded in RFT, skills denote trained response repertoires, such as defusion, acceptance, perspective taking, and committed action, which are explicitly taught and practiced in therapy (Hayes et al., 2006). In flow theory, skill denotes the person’s perceived capacity to meet the challenges perceived in a given activity: it is meaningful only in relation to those challenges and derives from the daily person-environment interaction rather than constituting a trained therapeutic repertoire (Massimini and Delle Fave, 2000; Nakamura and Csikszentmihalyi, 2002). Throughout the paper, therefore, therapy skills or flexibility skills refer to therapy, and skill without qualification refers to flow.

3 Note that through experience sampling methodologies, flow experience can be measured as it unfolds in real time, rather than inferred backwards from the behaviour it is meant to account for.

4 Note that this is a translation between vocabularies, not a claim that one level explains the other.

5 In ACT, defusion is best understood as a functional process, a shift in how a person relates to their own thoughts, rather than a single technique, even when it is trained through particular exercises (Assaz et al., 2018).
